# Predictive significance of arachidonate 15-lipoxygenase for eosinophilic chronic rhinosinusitis with nasal polyps

**DOI:** 10.1186/s13223-020-00480-8

**Published:** 2020-09-16

**Authors:** Zhuoping Liang, Bing Yan, Chang Liu, Ruyu Tan, Chengshuo Wang, Luo Zhang

**Affiliations:** 1 Department of Otolaryngology, Head and Neck Surgery, Beijing TongRen Hospital, Capital Medical University, Beijing, 100730 People’s Republic of China; 2 Beijing Key Laboratory of Nasal Diseases, Beijing Institute of Otolaryngology, Beijing, 100005 People’s Republic of China; 3 Department of Allergy, Beijing TongRen Hospital, Capital Medical University, Beijing, 100730 People’s Republic of China; 4 Research Unit of Diagnosis and Treatment of Chronic Nasal Diseases, Chinese Academy of Medical Sciences, Beijing, 100005 People’s Republic of China

**Keywords:** Arachidonate 15-lipoxygenase, Biomarker, Chronic rhinosinusitis with nasal polyps, Eosinophils, Predictive value, Receiver operating characteristic curve

## Abstract

**Background:**

Eosinophilic chronic rhinosinusitis with nasal polyps (ECRSwNP) exhibits a poorer outcome compared with non-eosinophilic chronic rhinosinusitis with nasal polyps (nonECRSwNP), so it is significant to identify effective markers to differentiate ECRSwNP in guiding the treatment strategies of these patients. Although arachidonate 15-lipoxygenase (ALOX15) is positioned as a marker of eosinophilic inflammation, its study in differentiating ECRSwNP has not been reported. The aim of this study is to assess the potential of ALOX15 in distinguishing and predicting ECRSwNP.

**Methods:**

Forty-eight patients with chronic rhinosinusitis with nasal polyps (CRSwNP), including 30 ECRSwNP and 18 nonECRSwNP patients, were enrolled. ALOX15 mRNA level was determined in polyps by real-time polymerase chain reaction (RT-PCR). The patients’ baseline characteristics were evaluated and analyzed for correlations with ALOX15. Receiver operating characteristic (ROC) curve was used to assess the predictive significance of the potential predictors for ECRSwNP.

**Results:**

ALOX15 mRNA level was significantly higher in ECRSwNP patients than in nonECRSwNP patients (*P* < 0.001). ALOX15 mRNA was significantly correlated with tissue and blood eosinophil percentages (*r* = 0.565, *P* < 0.001 and *r* = 0.395, *P* = 0.006), olfaction scores (*r* = 0.400, *P* = 0.005), total visual analogue scale (VAS) symptom scores (*r* = 0.383, *P* = 0.007), ethmoid/maxillary sinus (E/M) ratio (*r* = 0.463, *P* = 0.001), and endoscopy scores (*r* = 0.409, *P* = 0.004). Logistic regression analysis showed ALOX15 mRNA level and percentage of blood eosinophils to be predictive factors for ECRSwNP (*P* = 0.004 and *P* = 0.036, respectively). ROC curve indicated ALOX15 to have high predictive accuracy for ECRSwNP (area under the curve (AUC) = 0.909), which was further improved by combination of ALOX15 with percentage of blood eosinophils (AUC = 0.933).

**Conclusions:**

The relative ALOX15 mRNA level alone or in combination with blood eosinophils might be a reliable biomarker for predicting a diagnosis of ECRSwNP.

## Introduction

Chronic rhinosinusitis with nasal polyps (CRSwNP) is a spectrum of heterogeneous diseases. Based on the degree of infiltrating eosinophils, CRSwNP is subclassified into eosinophilic CRSwNP (ECRSwNP) and non-eosinophilic CRSwNP (nonECRSwNP) [1–3]. ECRSwNP represents a more aggressive phenotype characterized by a higher degree of disease severity, poorer responses to therapeutic interventions and higher polyp recurrence rates after surgery [4–6].

Given that ECRSwNP and nonECRSwNP potentially exhibit different responses to therapeutic interventions, distinguishing ECRSwNP from nonECRSwNP prior to surgery may be important in guiding the options for treatment [7]. In this regard, specific biomarkers are more objective in analyzing disease phenotype and the underlying pathogenesis [8], compared with endoscopic examinations, imaging techniques, and clinical characteristics.

Arachidonate 15-lipoxygenase (ALOX15), a member of the lipoxygenase family that metabolizes arachidonic acid (AA) to 15(S)-hydroperoxy-eicosatetraenoic acid (15-(S)-HETE) [9], is mainly expressed in eosinophils, activated monocytes, epithelial cells and mast cells [10]. ALOX15 has been suggested to promote eosinophil infiltration in eosinophilic inflammatory diseases, such as aspirin-exacerbated respiratory disease [11], eosinophilic esophagitis [12], and asthma [13]. Several studies have demonstrated increased levels of ALOX15 and its metabolites in asthma [14–16], which can lead to bronchial epithelial injury and remodeling [16] and regulate goblet cell differentiation in asthmatic human airway epithelial cells [9]. More recently, we have demonstrated that the expression of ALOX15 is significantly higher in patients with ECRSwNP than in patients with nonECRSwNP and healthy controls [17].

Considering the role of ALOX15 in eosinophilic inflammation, we hypothesized that ALOX15 may be a useful biomarker for differentiating ECRSwNP from nonECRSwNP. Thus, in this study, we aimed to evaluate the potential of ALOX15 alone or in combination with other characteristics in accurately predicting a diagnosis of ECRSwNP.

## Methods

### Subjects and tissue samples

A total of 48 subjects were enrolled from a consecutive population of CRSwNP patients undergoing endoscopic sinus surgery at Beijing TongRen Hospital, Capital Medical University from October 2016 to December 2017. The diagnoses of CRSwNP, comorbid asthma, allergic rhinitis, and atopy were made according to our previous report [18]. The patients were further classified as either ECRSwNP (n = 30) or non-ECRSwNP (n = 18) patients, based on the percentage of polyp tissue eosinophils being > 27% or ≤ 27% of the total infiltrating cells, respectively, according to the previous study [6]. Polyp tissues from all subjects were collected as biopsy specimens or during surgery for further analyses of eosinophils and ALOX15 mRNA levels as indicated below.

None of the subjects had received steroids, antibiotics, immunotherapy or anti-leukotrienes within 4 weeks before surgery. Patients with established fungal sinusitis, immunodeficiency, cystic fibrosis, or primary ciliary dyskinesia were excluded.

This study was approved by the ethics committee of Beijing TongRen Hospital, Capital Medical University, and written informed consent was obtained from all patients prior to enrolment into the study.

### Symptom score

The symptoms including nasal obstruction, rhinorrhea, olfaction, and facial pain or headache were assessed preoperatively using a visual analogue scale (VAS) ranging from 0 to 10 (0 represents the absence of any symptom and 10 represents the most severe symptom) as described previously [19]. The sum of these four VAS scores was calculated as total VAS symptom score.

### Computed tomography (CT) score

The CT score was evaluated by preoperative CT scan using the Lund-Mackay scoring system [20]. Each sinus group and the ostiomeatal complex were assigned a numeric grade: 0 = no abnormality, 1 = partial opacification, and 2 = total opacification, to provide a total CT score ranging from 0 to 24. The maxillary sinus score (M score), anterior ethmoid sinus score (AE score), and posterior ethmoid sinus score (PE score) were used to additionally calculate E (AE score + PE score) / M (M score) ratio.

### Endoscopy score

The endoscopy score for nasal polyps (NPs) was calculated for each nasal cavity by nasal endoscopy as previously described [21]. Each nasal cavity was graded as follows: 0 = no polyps; 1 = small polyps in the middle meatus not reaching below the inferior border of the middle concha; 2 = polyps reaching below the lower border of the middle turbinate; 3 = large polyps reaching the lower border of the attachment of inferior turbinate or polyps medial to the middle concha; 4 = large polyps causing almost complete congestion/obstruction of the inferior meatus. The sum of the nasal polyp scores for both sides was determined as the endoscopy score.

### Blood parameters

Blood samples were collected preoperatively and used to assess the blood routine parameters as well as sIgE levels for common aeroallergens including house dust mites, molds, trees, weed and grass pollen, and animal dander. All sIgE levels were detected using ImmunoCAP (Phadia, Uppsala, Sweden; cut-off value, 0.35 kUA/L).

### Hematoxylin and eosin (H&E) staining

Hematoxylin and eosin (H&E) staining was performed as previously described [17]. The stained sections were evaluated for eosinophil infiltration by an investigator blinded to the patients’ clinical characteristics, in 5 non-overlapping high-power fields (HPF, × 400 magnification) using a Leica microscope (Leica Microsystems, Bannockburn, USA).

The tissue eosinophils were expressed as a percentage of total infiltrating cells as follows: ${\text{Tissue eosinophils }}\left( \% \right) = \left( {{\text{number of tissue eosinophils }}/{\text{ HPF}}} \right) \times { }100{\text{\% }}/{\Sigma }({\text{number of tissue eosinophils}} + {\text{ number of tissue neutrophils}} + {\text{ number of tissue lymphocytes}} + {\text{ number of tissue plasma cells}})/{\text{HPF}}.$

### Real-time polymerase chain reaction (RT-PCR) analysis

Reverse transcription and RT-PCR were performed to detect ALOX15 mRNA level in nasal polyp tissues as previously described [17]. Primer sequences employed for RT-PCR were as follows: ALOX15, forward primer: GGGCAAGGAGACAGAACTCAA, reverse primer: CAGCGGTAACAAGGGAACCT; GAPDH, forward primer: 5′-CTCCTCCTGTTCG- ACAGTCAGC-3′, reverse primer: 5′-CCCAATACGACCAAATCCGTT-3′. The relative ALOX15 mRNA level was calculated according to the formula: 2^−ΔCt^ = 2^−(Ct ALOX15 − Ct GAPDH)^. For statistical analysis, 2^−ΔCt^ values were log_2_ (equivalent to -ΔCt) converted into linearization, which represented that the relative level of ALOX15 mRNA was normalized to GAPDH mRNA. A higher value of –ΔCt represents a higher level of ALOX15 mRNA expression.

### Statistical analysis

Statistical analysis was performed using SPSS 23.0 (IBM Corp, Armonk, USA). Values of the continuous normal distribution were presented as mean ± standard deviation, others were expressed as median and interquar range. Continuous variable differences and unpaired comparisons were analyzed by the Student’s *t*-test or Mann–Whitney *U* test, respectively. Sex, previous surgery, asthma, allergic rhinitis and atopy were compared using Chi-square test. Correlations between ALOX15 mRNA expression and laboratory and clinical characteristics were assessed by Spearman correlation test. Binary logistic regression was used to detect potential predictors for ECRSwNP. The predictive ability of ALOX15 and other predictors was evaluated by receiver operating characteristic (ROC) curve. MedCalc statistical software (version 15.2, Ostend, Belgium) was used to compare AUC levels of the factors. *P* < 0.05 was considered statistically significant.

## Results

### Comparison of the demographic, laboratory and clinical characteristics between ECRSwNP and nonECRSwNP

**Fig. 1:**
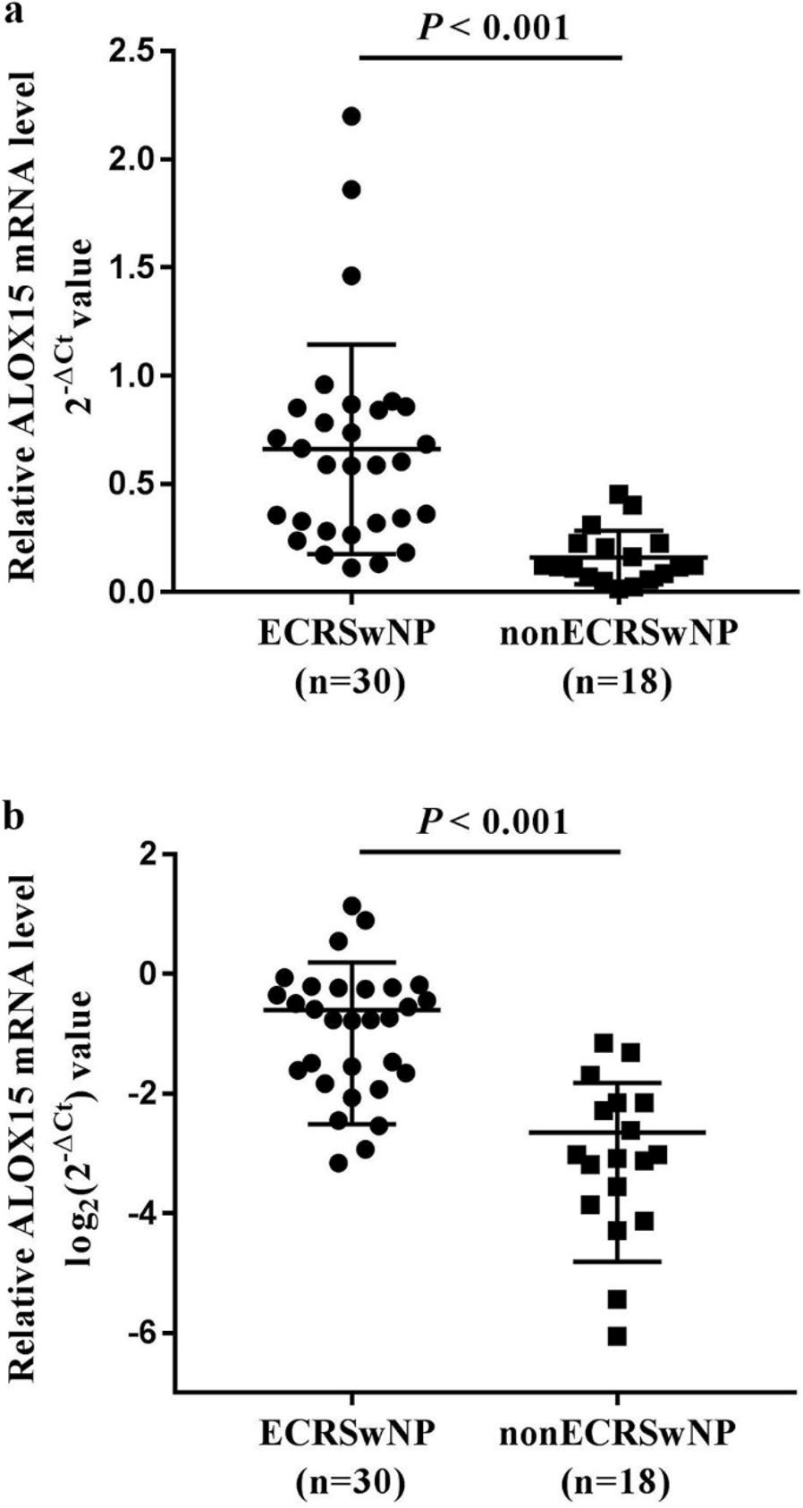
Relative expression of ALOX15 mRNA levels in nasal mucosa tissues of ECRSwNP and nonECRSwNP determined by RT-PCR. **a** The relative expression level of ALOX15 mRNA was determined by 2^−ΔCt^ value. **b** The relative mRNA level of ALOX15, which corresponded to the level of -ΔCt, was analyzed by log_2_(2^−ΔCt^) methods. A higher -ΔCt value represents a higher ALOX15 mRNA level. *ALOX15* arachidonate 15-lipoxygenase, *ECRSwNP* eosinophilic chronic rhinosinusitis with nasal polyps, *nonECRSwNP* non-eosinophilic chronic rhinosinusitis with nasal polyps, *RT-PCR* real-time polymerase chain reaction

Thirty CRSwNP patients (62.5%) were classified into the eosinophilic group, and eighteen CRSwNP patients (37.5%) were classified into non-eosinophilic group. Significant differences were noted in the levels of tissue and blood eosinophils (all *P* < 0.001), tissue neutrophils (*P* = 0.003), olfaction scores (*P* = 0.002), total VAS scores (*P* = 0.021), E/M ratio (*P* = 0.003), and endoscopy scores (*P* = 0.003) between the two groups (Table 1). Then, ECRSwNP patients were more likely to have asthma (*P* = 0.003), allergic rhinitis (*P* = 0.044) and atopy (*P* = 0.034) (Table 1), as well as higher ALOX15 mRNA levels in nasal polyp tissues (*P* < 0.001) (Fig. 1). However, the two groups were not significantly different with other characteristics (shown in Table 1).

**Table 1:** Baseline characteristics of patients

	ECRSwNP (n = 30)	NonECRSwNP (n = 18)	*P* value
Demographic characteristics			
Sex, male/female	17/13	11/7	0.762
Age, y (mean ± SD)	45.03 ± 11.86	46.00 ± 15.77	0.810
Previous surgery, n (%)	10 (33.33)	7 (38.89)	0.697
Asthma, n (%)	16 (53.33)	2 (11.11)	*0.003*
Allergic rhinitis, n (%)	9 (30.00)	1 (5.56)	*0.044*
Atopy, n (%)	16 (53.33)	4 (22.22)	*0.034*
Histopathological findings (H&E staining)			
Tissue eosinophils (%), median (IQR)	57.27 (41.86–68.43)	4.19 (1.97–6.68)	* < 0.001*
Tissue neutrophils (%), median (IQR)	0.00 (0.00–2.18)	6.84 (2.53–13.39)	*0.003*
Self-assessed symptom scores (VAS)			
Nasal obstruction score, median (IQR)	7.00 (6.00–8.00)	6.00 (6.00–8.00)	0.572
Rhinorrhea score, median (IQR)	6.00 (5.00–7.00)	5.50 (0.00–6.00)	0.078
Olfaction score, median (IQR)	9.00 (6.00–10.00)	6.00 (4.00–8.00)	*0.002*
Facial pain or headache score, median (IQR)	0.00 (0.00–5.00)	0.00 (0.00–5.25)	0.581
Total VAS score, median (IQR)	22.50 (19.00–25.50)	19.50 (16.75–22.25)	*0.021*
Imaging examination scores			
CT score, median (IQR)	17.50 (14.00–22.00)	17.50 (11.00–20.25)	0.326
E/M ratio, median (IQR)	2.33 (2.00–3.50)	2.00 (2.00–2.00)	*0.003*
Endoscopy score, median (IQR)	5.50 (4.00–7.00)	4.00 (2.75–5.00)	*0.003*
Routine blood tests			
Blood eosinophils (%), median (IQR)	6.25 (5.05–9.20)	1.60 (0.78–4.95)	* < 0.001*
Blood neutrophils (%), median (IQR)	53.45 (47.03–56.68)	58.50 (48.18–69.75)	0.058

*ECRSwNP* eosinophilic chronic rhinosinusitis with nasal polyps, *nonECRSwNP* non-eosinophilic chronic rhinosinusitis with nasal polyps, *y* years, *SD* standard deviation, *n* number, *H&E* hematoxylin and eosin, *IQR* interquartile range, *VAS* visual analogue scale, *CT* computed tomography, *E/M* ethmoid/maxillary sinus. Italic values are significant at *P* < 0.05

### Associations between ALOX15 level and laboratory and clinical characteristics

The level of ALOX15 mRNA was significantly correlated with tissue eosinophils (*r* = 0.565, *P* < 0.001), olfaction scores (*r* = 0.400, *P* = 0.005), total VAS scores (*r* = 0.383, *P* = 0.007), E/M ratio (*r* = 0.463, *P* = 0.001), endoscopy scores (*r* = 0.409, *P* = 0.004), and blood eosinophils (*r* = 0.395, *P* = 0.006); but not with age, tissue neutrophils, nasal obstruction scores, rhinorrhea scores, facial pain or headache scores, CT scores, or blood neutrophils (shown in Table 2).

**Table 2:** Spearman correlations between ALOX15 mRNA level and laboratory and clinical characteristics

Correlation		*r* value	*P* value
ALOX15 mRNA level (− ΔCt value)	Age	− 0.116	0.433
	Tissue eosinophils (%)	0.565	* < 0.001*
	Tissue neutrophils (%)	− 0.167	0.256
	Nasal obstruction score	0.149	0.312
	Rhinorrhea score	0.225	0.124
	Olfaction score	0.400	*0.005*
	Facial pain or headache score	0.056	0.706
	Total VAS score	0.383	*0.007*
	CT score	0.150	0.309
	E/M ratio	0.463	*0.001*
	Endoscopy score	0.409	*0.004*
	Blood eosinophils (%)	0.395	*0.006*
	Blood neutrophils (%)	− 0.159	0.280

*ALOX15* arachidonate 15-lipoxygenase, *VAS* visual analogue scale, *CT* computed tomography, *E/M* ethmoid/maxillary sinus. Italic values are significant at *P* < 0.05

### Assessment of the predictive value of the ALOX15 mRNA for ECRSwNP

To determine the predictive value of the ALOX15 mRNA for ECRSwNP, binary logistic regression analysis was conducted. Blood eosinophils, which has been shown to predict ECRSwNP previously [18], has also been introduced. The regression indicated that both ALOX15 mRNA (OR = 4.440, 95% CI = 1.614—12.213, *P* = 0.004) and blood eosinophils (OR = 1.457, 95% CI = 1.024—2.074, *P* = 0.036) showed the potential in predicting ECRSwNP (Table 3), and were thus, further subjected to ROC analysis.

**Table 3:** Binary logistic regression analysis for potential factors predicting ECRSwNP

Factors	OR	95% CI	*P* value
ALOX15 (− ΔCt value)	4.440	1.614–12.213	0.004
Blood eosinophils (%)	1.457	1. 024–2.074	0.036

*ECRSwNP* eosinophilic chronic rhinosinusitis with nasal polyps, *OR* odds ratio, *CI* confidence interval, *ALOX15* arachidonate 15-lipoxygenase

**Fig. 2:**
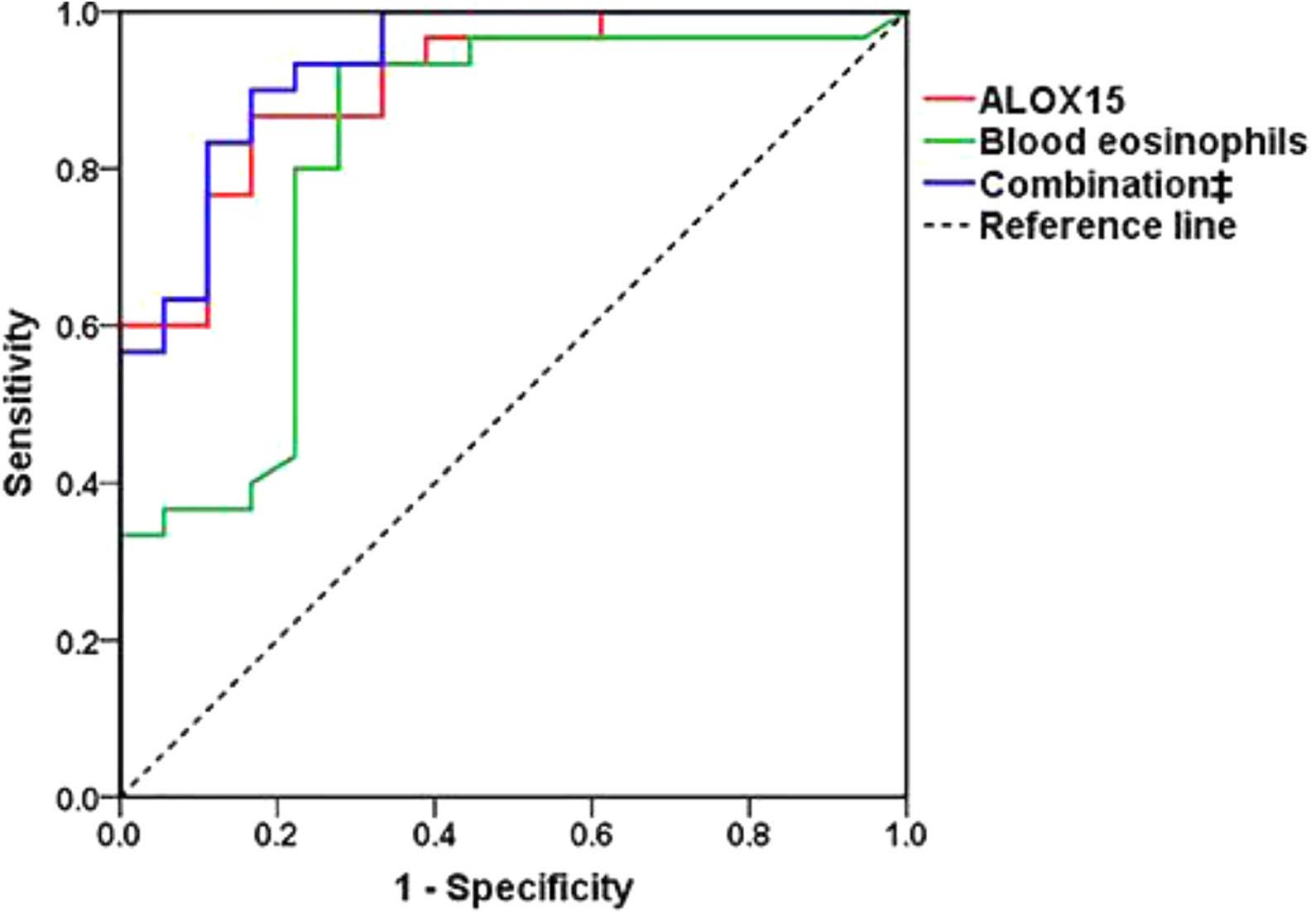
ROC curves of ALOX15, blood eosinophils, and their combination for predicting the diagnosis of ECRSwNP. The predictive ability was calculated according to AUC. ALOX15 alone and in combination with blood eosinophils demonstrated high accuracies, and blood eosinophils alone moderate accuracy in predicting ECRSwNP (AUC: 0.909, 0.933, and 0.820, respectively). *ROC* receiver operator characteristic, *ALOX15* arachidonate 15-lipoxygenase, *ECRSwNP* eosinophilic chronic rhinosinusitis with nasal polyps, *AUC* area under the curve. ‡: combined ALOX15 with blood eosinophils

ROC curve analysis showed that ALOX15 mRNA had a high accuracy, and blood eosinophils a moderate accuracy as predictors of ECRSwNP (AUC = 0.909, 95% CI = 0.828—0.990, *P* < 0.001; and AUC = 0.820, 95% CI = 0.687—0.954, *P* < 0.001, respectively) (Fig. 2). A maximal Youden index value of 0.700 demonstrated a cut-off value of -2.113 for ALOX15 to predict the diagnosis of ECRSwNP with a sensitivity of 83.3% and a specificity of 86.7% (Fig. 2). Similarly, a maximal Youden index value of 0.656 demonstrated a cut-off point of 3.45% for blood eosinophils in predicting the diagnosis of ECRSwNP with a sensitivity of 72.2% and a specificity of 93.3% (Fig. 2).

In view of the slightly lower accuracy and sensitivity of blood eosinophils in predicting ECRSwNP, we speculated that a combination of ALOX15 and blood eosinophils might improve the value of blood eosinophils in predicting ECRSwNP. A model comprising these combined predictors, derived from logistic regression analysis, was thus established as follows:

Model = ALOX15 mRNA level (-ΔCt value) + blood eosinophils (%) × 37.7/1.491.

ROC curve analysis of the combined data for ALOX15 and blood eosinophils indicated an increased AUC of 0.933; with a cut-off value of -0.778 and sensitivity of 83.3% and specificity of 90.0%; which were improvements over the accuracy and sensitivity of blood eosinophils, as well as the specificity of ALOX15 mRNA. Although the AUC for the combination of ALOX15 and blood eosinophils was not significantly different from the AUC of ALOX15 mRNA (*P* = 0.363) alone, this was statistically different from the AUC of blood eosinophils (*P* = 0.028); indicating a higher value of the combination of ALOX15 mRNA and blood eosinophils in predicting ECRSwNP, than the value of blood eosinophils alone.

### Comparisons of the laboratory and clinical characteristics between the high- and the low-combination level group

**Fig. 3:**
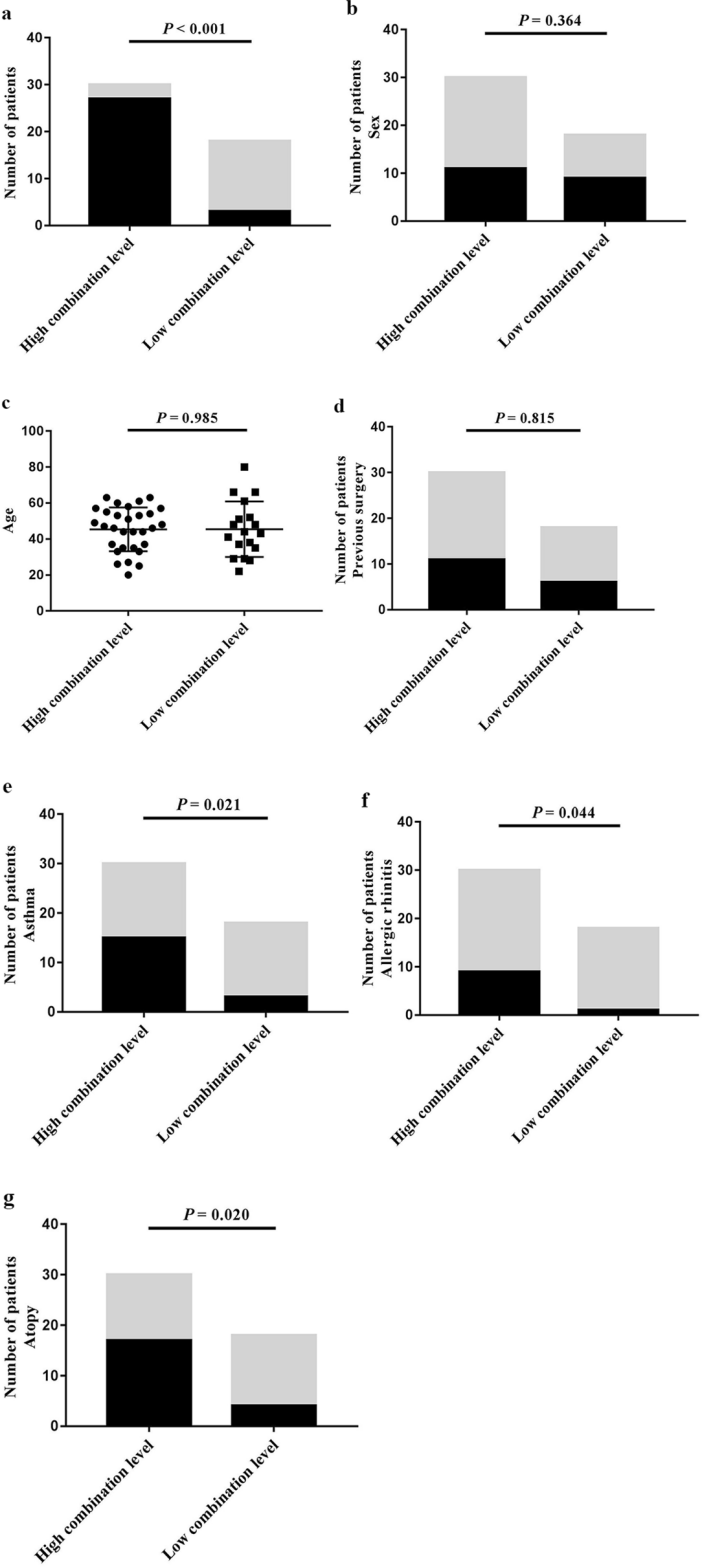
Comparison of the demographic characteristics between the high combination level group and the low combination level group, divided according to the predictive cut-off value. **a** number of ECRSwNP patients (black columns) / nonECRSwNP patients (grey columns), **b** sex (black columns for female, grey columns for male), **c** age, **d** previous surgery, **e** comorbid asthma, **f** comorbid allergic rhinitis, and **g** comorbid atopy (**d**-**g**, black columns for yes and grey columns for no for other characteristics). *ECRSwNP* eosinophilic chronic rhinosinusitis with nasal polyps, *nonECRSwNP* non-eosinophilic chronic rhinosinusitis with nasal polyps. The combination represents arachidonate 15-lipoxygenase with blood eosinophils

Based on the optimal cut-off value of -0.778 for the combination of ALOX15 mRNA and blood eosinophils, the CRSwNP patients were divided into a high combination level group (values ≥ -0.778, n = 30) and a low combination level group (values < -0.778, n = 18). The occurrence of ECRSwNP was significantly higher in the high combination level group (n = 27) than in the low combination level group (n = 3) (*P* < 0.001) (Fig. 3a).

**Fig. 4:**
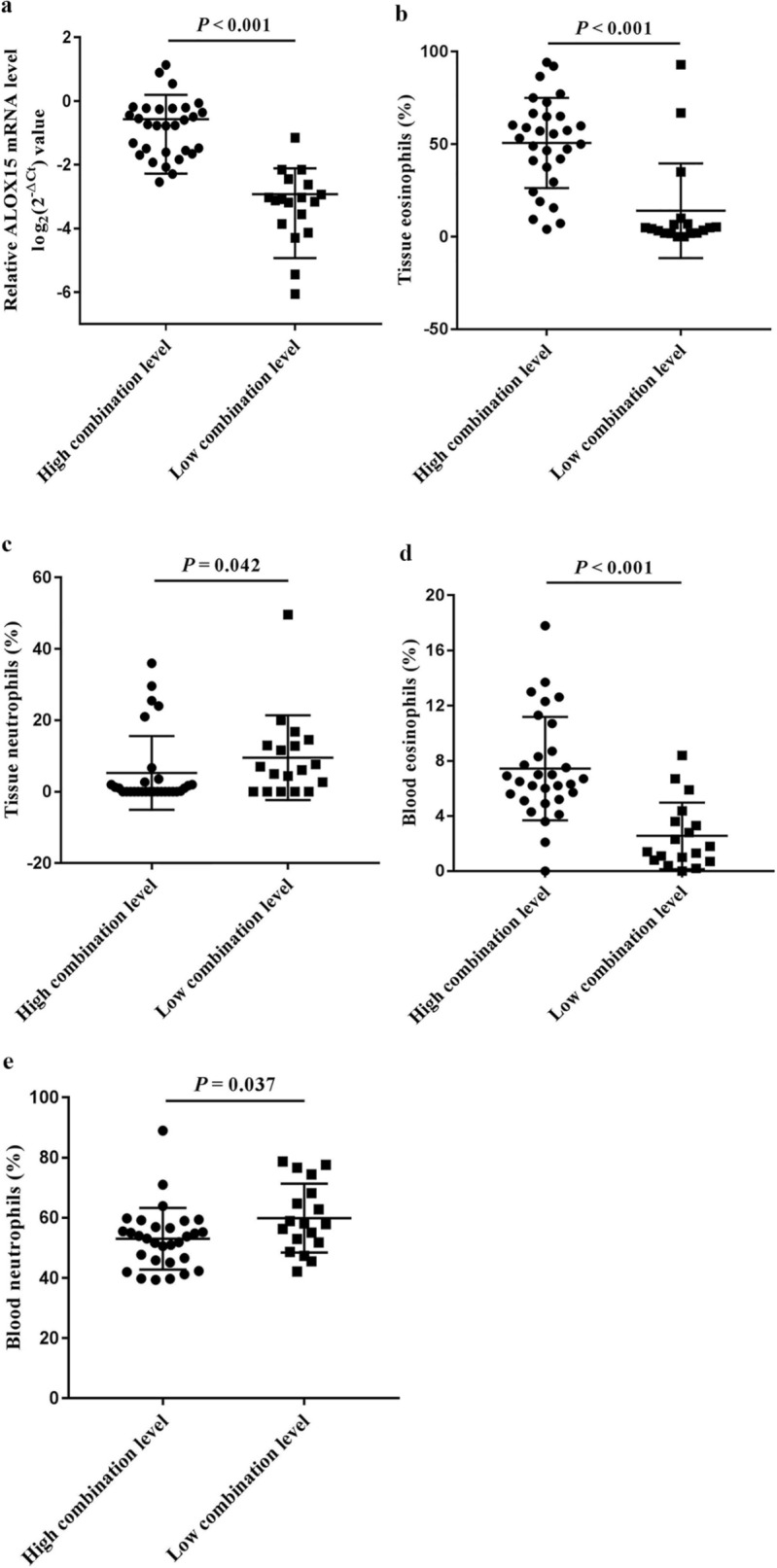
Infiltration of inflammatory cells in nasal polyp tissues and peripheral blood, and ALOX15 mRNA level in CRSwNP patients with the combination level greater than or less than the predictive cut-off value. **a** ALOX15 mRNA (-ΔCt value), **b** tissue eosinophils, **c** tissue neutrophils, **d** blood eosinophils, and **e** blood neutrophils. *ALOX15* arachidonate 15-lipoxygenase, *CRSwNP* chronic rhinosinusitis with nasal polyps. The combination represents arachidonate 15-lipoxygenase with blood eosinophils

**Fig. 5:**
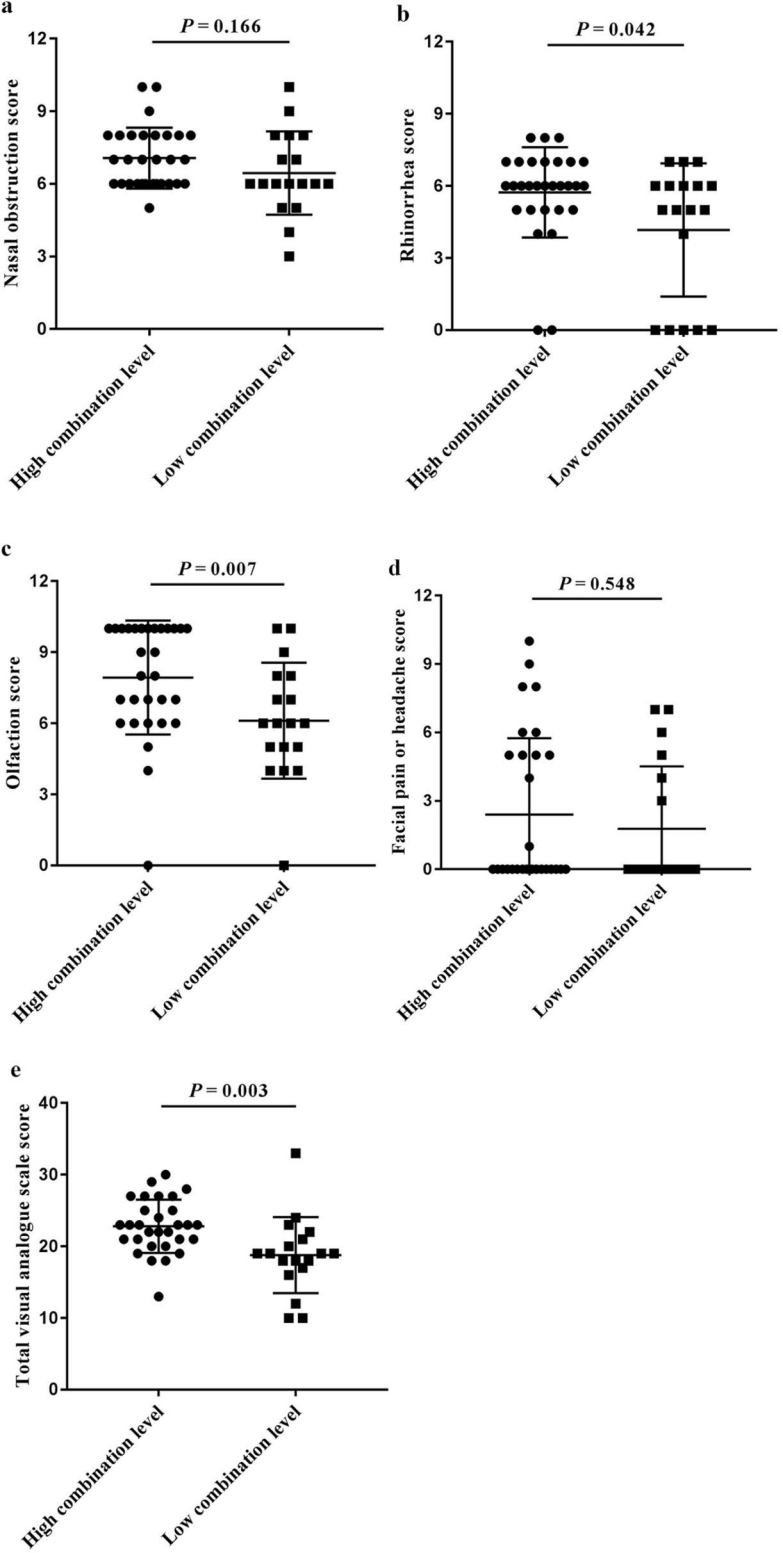
Comparison of self-assessed symptom scores (VAS) between the high combination level group and the low combination level group divided according to the predictive cut-off value. **a** nasal obstruction score, **b** rhinorrhea score, **c** olfaction score, **d** facial pain or headache score, and **e** total VAS score. *VAS* visual analogue scale. The combination represents arachidonate 15-lipoxygenase with blood eosinophils

**Fig. 6:**
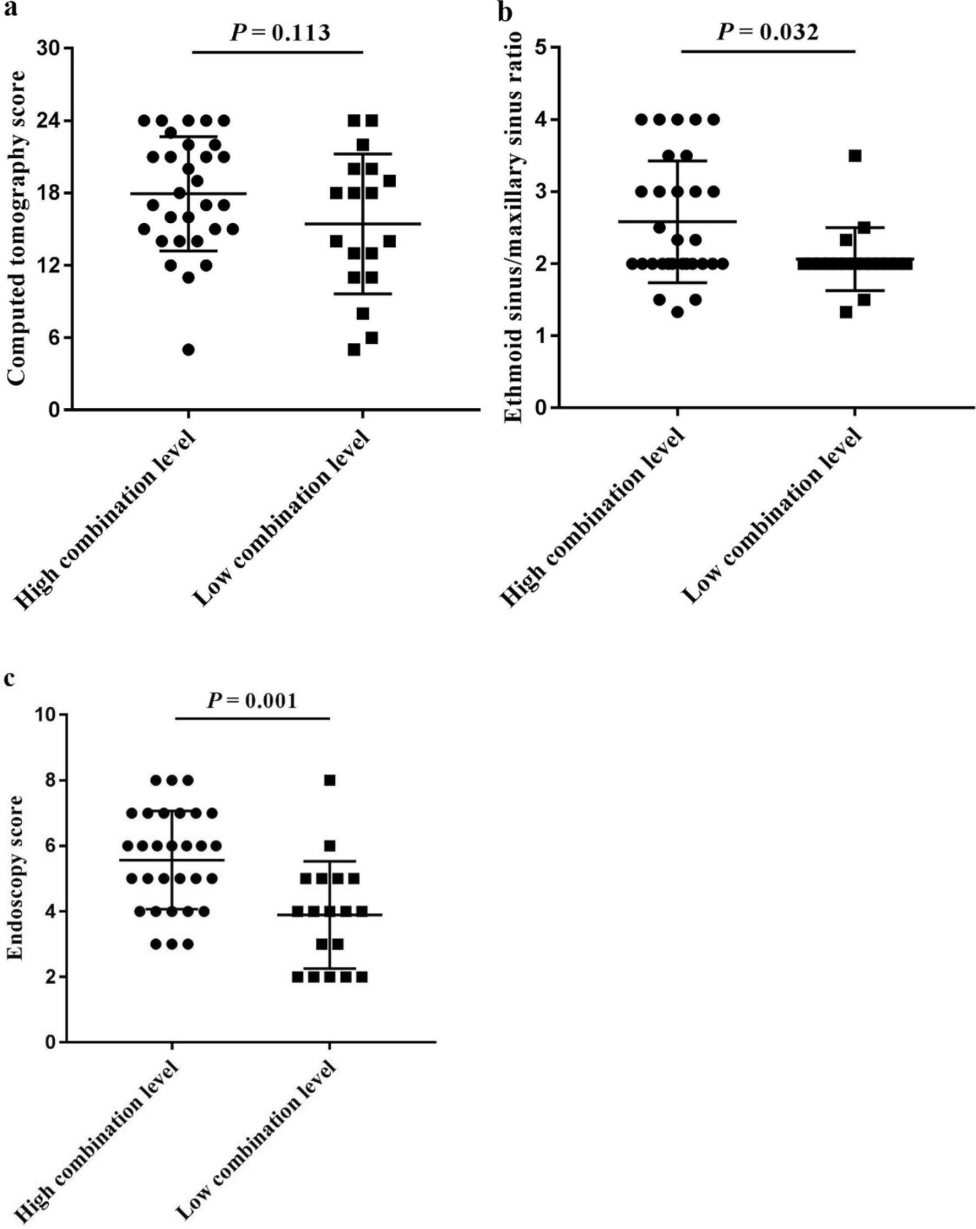
Comparison of the imaging examination scores between the high combination level group and the low combination level group divided according to the predictive cut-off value. **a** CT score, **b** E/M ratio, and **c** Endoscopy score. *CT* computed tomography, *E/M* ethmoid/maxillary sinus. The combination represents arachidonate 15-lipoxygenase with blood eosinophils

Comparison of the demographic and clinical characteristics of the two groups demonstrated that the combined level was not significantly associated with the distribution of sex, age, previous surgery (Fig. 3b-d). However, patients in the high combination level group were more likely to suffer from asthma (*P* = 0.021), allergic rhinitis (*P* = 0.044), and atopy (*P* = 0.020) (Fig. 3e-g). Moreover, patients with high combination levels showed significantly higher ALOX15 mRNA levels and higher eosinophil, but not neutrophil infiltration, in both polyp tissue and peripheral blood than patients with low combination levels (all *P* < 0.001) (Fig. 4). Assessment of self-assessed symptom scores demonstrated that rhinorrhea scores (*P* = 0.042), olfaction scores (*P* = 0.007), and total VAS scores (*P* = 0.003) were significantly higher in patients with high combination levels (Fig. 5). Similarly, analysis of imaging examination scores indicated that E/M ratio (*P* = 0.032) and endoscopy scores (*P* = 0.001), but not CT scores, were significantly higher in patients with high combination levels (Fig. 6).

## Discussion

Compared with nonECRSwNP, ECRSwNP has a closer relationship to airway disease such as asthma with broader eosinophilic airway inflammation [22, 23], and tends to have higher incidence rates of asthma and allergic rhinitis [24–26]. Indeed, our findings for the higher incidence rates for asthma, allergic rhinitis and atopy in ECRSwNP patients compared with nonECRSwNP patients in the present study are consistent with the findings of these earlier studies. Then, as patients with ECRSwNP have a greater chance of relapse after surgery, increased rates of postsurgical medication, and poorer quality of life than patients with non-eosinophilic or neutrophilic phenotypes [5, 6, 27–30]. Thus, to distinguish CRSwNP as eosinophilic or non-eosinophilic CRSwNP is likely to be important for formulating individualized treatment plans, predicting the prognosis, and reducing recurrence.

Currently, several clinical characteristics such as tissue eosinophils [1, 6], blood eosinophils [31], E/M ratio [24], and the Japanese Epidemiological Survey of Refractory Eosinophilic Chronic Rhinosinusitis (JESREC) [32] scores are used to identify ECRSwNP. In the present study, we evaluated the significance of clinical characteristics to differentiate between ECRSwNP and nonECRSwNP. In accordance with findings from other studies [32–34], our study also showed significant differences between the two sub-phenotypes, with respect to symptom scores, E/M ratio, endoscopy scores and blood eosinophils.

Unlike clinical characteristics, biomarkers are considered to be more objective indicators in evaluating ECRSwNP. Some studies have shown Charcot-Leyden crystals (CLCs) to be a marker of eosinophils [35, 36], and studies from our group have demonstrated CLCs to be a predictor of ECRSwNP [18, 37]. Unlike CLCs, ALOX15 as a metabolic enzyme, which together with its metabolites plays an important role in eosinophilic inflammation and influences epithelial cell function at a local metabolic level [9, 13, 15, 17, 38–45]. More recently, a genome-wide association study involving nearly 10,000 NP and CRS patients and over 70,000 controls has indicated a missense variant in ALOX15 that causes alteration in enzymatic activity and confers large genome-wide significant protection against NP [46]. In our study, we found that ALOX15 mRNA level was significantly increased in ECRSwNP patients compared with nonECRSwNP patients and positively associated with tissue eosinophils, olfaction scores, total VAS scores, E/M ratio, endoscopy scores, and blood eosinophils; suggesting that ALOX15 might have predictive significance for ECRSwNP.

Indeed, analysis of ALOX15 mRNA and blood eosinophils by a binary logistic regression model and ROC curve analysis, suggested that ALOX15 might be a valuable predictor of ECRSwNP as shown by high accuracy, whereas by comparison, blood eosinophils had lower accuracy in predicting ECRSwNP. Moreover, the finding that combination of ALOX15 mRNA and blood eosinophils could improve the accuracy and sensitivity of blood eosinophils in predicting for ECRSwNP indicates that combining the two factors has an optimal predictive value for ECRSwNP. These results also suggest that if patients with ECRSwNP present with both ALOX15 > -2.113 and blood eosinophils > 3.45%, then greater attention needs to be paid to these patients and more active and effective treatment measures need to be implemented for these patients in clinical practice.

Furthermore, analysis of ECRSwNP patients based on the optimal cut-off value of -0.778 for the combination of ALOX15 mRNA and blood eosinophils, the CRSwNP patients with values ≥ -0.778 (high combination group) had significantly higher comorbid asthma, allergic rhinitis, atopy, rhinorrhea scores, olfaction scores, total VAS scores, E/M ratio, endoscopy scores, and blood eosinophils compared with CRSwNP patients with values < -0.778 (low combination group), which were basically consistent with the results of ECRSwNP group mentioned above. As eosinophilic CRS (ECRS) shares many histologic and immunologic features with asthma, it is possible that ECRS and asthma may be influenced by the same immune processes in the upper and lower airways [47–49]. The findings of the present study also support this view and suggest that ALOX15 probably plays an important pathophysiologic role in both the upper and lower airways. Thus, according to the concept of “one airway, one disease”, patients with both ECRSwNP and asthma with increased ALOX15 expression in polyp tissues, would likely benefit from treatment for both diseases.

However, our study has some limitations, including the relatively small sample size and the role of ALOX15 in ECRSwNP was assessed only by determining the ALOX15 mRNA level. Moreover, only a preliminary inference has been made that ALOX15 might play a role in the upper and lower airways involving ECRSwNP and asthma. Therefore, large study sample size is necessary to further verify the predictive value of ALOX15 for ECRSwNP, and to validate the role of ALOX15 in ECRSwNP from multiple levels. In addition, the specific mechanism of ALOX15 in the upper and lower airway inflammation needs to be further studied in the future.

## Conclusions

In conclusion, the present study showed that the expression of ALOX15 was significantly upregulated in ECRSwNP patients, compared with nonECRSwNP patients. Furthermore, the level of ALOX15 mRNA expression was correlated with the numbers of tissue and blood eosinophils, olfaction scores, facial pain or headache scores, total VAS scores, E/M ratio, and endoscopy scores; and additionally exhibited a high accuracy for predicting ECRSwNP. Overall, these findings suggest that ALOX15 might be a reliable biomarker for differentiating ECRSwNP patients from nonECRSwNP patients and thus provide a new intervention target for the clinical treatment of ECRSwNP as well be important in guiding future treatment strategies for these patients.

## Data Availability

The datasets used and/or analyzed during the current study are available from the corresponding author on reasonable request.

## Abbreviations

ECRSwNP: Eosinophilic chronic rhinosinusitis with nasal polyps
NonECRSwNP: Non-eosinophilic chronic rhinosinusitis with nasal polyps
ALOX15: Arachidonate 15-lipoxygenase
CRSwNP: Chronic rhinosinusitis with nasal polyps
RT-PCR: Real-time polymerase chain reaction
ROC: Receiver operating characteristic
VAS: Visual analogue scale
E/M: Ethmoid/maxillary sinus
AUC: Area under the curve
AA: Arachidonic acid
15-(S)-HETE: 15(S)-hydroperoxy-eicosatetraenoic acid
N: Number
CT: Computed tomography
M score: Maxillary sinus score
AE score: Anterior ethmoid sinus score
PE score: Posterior ethmoid sinus score
NPs: Nasal polyps
H&E: Hematoxylin and eosin
HPF: High-power fields
CI: Confidence interval
JESREC: Japanese Epidemiological Survey of Refractory Eosinophilic Chronic Rhinosinusitis
CLCs: Charcot-Leyden crystals
ECRS: Eosinophilic chronic rhinosinusitis
Y: Years
SD: Standard deviation
IQR: Interquartile range
OR: Odds ratio
